# Optimising Return to Work for Cardiovascular Patients: An Interdisciplinary Approach in Occupational Medicine and Cardiology

**DOI:** 10.3390/life16010019

**Published:** 2025-12-22

**Authors:** Donatella Sansone, Antonella Cherubini, Fabiano Barbiero, Marina Bollini, Marcella Mauro, Andrea Di Lenarda, Francesca Rui, Luca Cegolon, Francesca Larese Filon

**Affiliations:** 1 UOC Unit of Occupational Medicine, Department of Medical Sciences, University of Trieste, 34129 Trieste, Italy; donatella.sansone@asugi.sanita.fvg.it (D.S.); mmauro@units.it (M.M.); 2 Cardiovascular Center, Azienda Sanitaria Universitaria Giuliano Isontina (ASUGI), 34122 Trieste, Italy; antonella.cherubini@asugi.sanita.fvg.it (A.C.); marina.bollini@asugi.sanita.fvg.it (M.B.); andredilenarda@asugi.sanita.fvg.it (A.D.L.); 3 Department of Medical, Surgical & Health Sciences, University of Trieste, 34129 Trieste, Italy; frui@units.it (F.R.); luca.cegolon@units.it (L.C.)

**Keywords:** return to work, occupational medicine, cardiovascular disease, cardiac rehabilitation, quality of life, work ability

## Abstract

**Background:** This study explored facilitators and barriers to return to work (RTW) after acute cardiovascular events or elective cardiac surgery, integrating clinical, functional, and occupational factors. **Methods:** A prospective cohort study was conducted at the Occupational Medicine and Cardiac Rehabilitation Units of the Maggiore Hospital in Trieste, Italy. Employed adults (18–67 years) admitted for acute coronary syndrome, valve replacement, or thoracic aortic surgery between January 2024 and July 2025 were enrolled. Sociodemographic, clinical, and occupational data were collected alongside functional and psychosocial assessments, including the Work Ability Index (WAI) and EQ-5D-5L. Predictors of RTW were analyzed with Cox regression models. **Results:** Among 103 patients (mean age 56.8 years; 92.2% male), 77.7% returned to work after a mean of 58.9 days. Independent predictors of earlier RTW were self-employment (HR 5.08, 95% CI 2.52–10.27), occupational responsibility (HR 2.12, 95% CI 1.01–4.45), and percutaneous coronary intervention (HR 2.72, 95% CI 1.47–5.06). Higher job-related physical demands, arrhythmias, and cardiac rehabilitation participation were associated with delayed RTW. Mean WAI (37.2 ± 5.1) and EQ-5D index (0.92 ± 0.09; EQ-VAS 77.4 ± 12.9) indicated preserved function and quality of life. **Conclusions:** RTW after cardiovascular events is multifactorial. Integrating occupational medicine into cardiac rehabilitation is key to ensuring safe, sustainable reintegration.

## 1. Introduction

Cardiovascular disease (CVD) remains the leading cause of morbidity and mortality worldwide, accounting for nearly one-third of all deaths [1,2]. Advances in primary and secondary prevention have markedly improved survival [3], resulting in a growing population of working-age adults living with chronic CVD after hospitalization for myocardial infarction, coronary revascularization, or valve surgery.

This epidemiological shift has major implications for occupational medicine, especially in the context of an aging workforce and delayed retirement. Beyond clinical recovery, return to work (RTW) represents a crucial component of cardiac rehabilitation (CR) [4,5]. It is not only an indicator of functional recovery but also a determinant of psychological well-being, social reintegration, and economic stability [6].

Historically, prolonged rest and early retirement were often recommended after myocardial infarction or major cardiac surgery. However, contemporary evidence supports early mobilization and occupational reintegration, leading to a paradigm shift in both clinical practice and patient expectations [7]. RTW is now considered an integral part of post-cardiac rehabilitation and a relevant outcome of CV care.

RTW outcomes after CV events are influenced by multiple and interrelated factors, as reported in previous studies [8,9]. These include clinical recovery and residual functional capacity [9,10], type and invasiveness of the intervention [11], participation in structured cardiac rehabilitation programs [12], and sociodemographic characteristics such as age and sex [13,14]. Occupational factors, including job demands, physical workload, and shift work, have also been shown to affect both the likelihood and timing of RTW [8,9]. In addition, psychosocial aspects—such as self-perceived work ability, social support, and anxiety related to recurrence—may play an important role in the reintegration process.

Despite its relevance, evidence on the determinants of RTW after CV events remains heterogeneous, and the combined influence of clinical, psychosocial, and occupational variables is not fully understood. Few studies in the literature have simultaneously integrated such a broad set of variables to assess their relative impact on RTW outcomes [4,5,15]. A deeper understanding of these interactions can guide personalized rehabilitation strategies and workplace adjustments aimed at sustainable reintegration.

The primary objective of this study was to identify facilitators and barriers to RTW among employed patients recovering from acute CV events or elective cardiac surgery. Specifically, we examined the timing of RTW and its associations with sociodemographic, clinical, and occupational characteristics, incorporating subjective measures of work ability and health-related quality of life.

## 2. Materials and Methods

### 2.1. Study Design and Participants

This prospective, observational cohort study was conducted at the Occupational Medicine and Cardiac Rehabilitation Units of Cardiovascular Center in the Azienda Sanitaria Universitaria Giuliano-Isontina (ASUGI) in Trieste, Italy, between January 2024 and July 2025.

Eligible participants were adults aged 18–67 years who were employed at the time of hospital admission for an acute CV event or elective cardiac surgery. Acute events included ST-segment elevation myocardial infarction (STEMI), non-ST-segment elevation myocardial infarction (NSTEMI), acute aortic syndrome and acute heart failure. Surgical or interventional procedures comprised percutaneous coronary intervention (PCI), coronary artery bypass grafting (CABG), valve replacement, and thoracic aortic surgery.

Patients were excluded if they were retired or unemployed before the index event, had severe cognitive impairment, or were unable to provide informed consent.

### 2.2. Data Collection

Data were obtained through structured interviews, clinical records, and occupational assessments. The following variables were collected:

• Sociodemographic: age, sex, smoking status (pre-admission and post-discharge), and family history of CVD.
• Clinical: body mass index (BMI), CV risk factors, Charlson Comorbidity Index (CCI), diagnosis of CVD, out-of-hospital cardiac arrest (OHCA) occurring before admission as a complication of the index acute event, type of intervention, periprocedural complications, echocardiographic parameters, arrhythmias (recorded at ECG, 24 h-Holter and telemetry), medications, and participation in CR. Left ventricular ejection fraction (LVEF) was assessed by echocardiography at discharge and during follow-up, and analyzed both as mean group values and as within-patient change to capture individual improvement over time. Exercise stress testing was performed to assess functional status and was designed to exceed patients’ occupational metabolic demands.
• Occupational: job type, employment status, working hours, shift work, exposure to occupational hazards, contract type, level of responsibility, and estimated task-specific metabolic equivalents (METs) according to the Compendium of Physical Activities [16].

RTW status, time to RTW, and any new work restrictions were recorded during follow-up.

### 2.3. Functional and Psychosocial Assessments

Two validated instruments were used:

• Work Ability Index (WAI): self-administered questionnaire evaluating perceived ability to meet work demands, taking into account physical and mental capacity, diagnosed diseases, and personal resources. Scores range from 7 (poor) to 49 (excellent) [17].
• EQ-5D-5L: standardized measure of health-related quality of life (HRQoL), comprising five dimensions (mobility, self-care, usual activities, pain/discomfort, anxiety/depression) and a Visual Analogue Scale (VAS) ranging from 0 (worst imaginable health) to 100 (best imaginable health) [18]. The Italian value set was applied to compute the EQ-5D index.

Fear of recurrence was evaluated indirectly by documenting emergency department visits post-discharge, considered a proxy for anxiety or perceived clinical illness.

### 2.4. Statistical Analysis

The main characteristics of our study population were described by mean, standard deviation (SD), and median in the case of continuous variables and by frequency and percentage in the case of categorical variables.

Comparisons of baseline characteristics between groups were performed using non-parametric tests: the Mann–Whitney U test or Kruskal–Wallis test for continuous variables, and the chi-square test of independence for categorical variables. We used Cox proportional hazards regression to model the association between selected predictors and the likelihood of RTW.

Candidate clinical and occupational predictors included age, BMI, diabetes, Charlson Comorbidity Index (CCI), type of treatment (PCI, CABG, VR, aortic surgery), arrhythmia post-discharge, CR, occupational Physical Demand, shift work, type of job contract (self-employed vs. employed), responsibility at work, Work Ability Index (WAI) and EQ visual analogue scale (EQ VAS). All were selected based on their relevance reported in the literature as potential determinants of RTW [5,8,19,20].

Starting from a multiple Cox regression model, a stepwise selection criterion was applied and only covariates with a *p*-value < 0.20 were included in the final model. The proportional hazards assumption was tested using scaled Schoenfeld residuals with the Grambsch–Therneau test [18]. No evidence of violation of the proportional hazards’ assumption was found [18]. Final model was stratified by sex, smoking status, out-of-hospital cardiac arrest and heart failure, to account for differences in baseline hazards. To provide intermediate evaluation, for each covariate included in our final model, univariate and bivariate models adjusted for age were also computed. Every return to work in the follow-up period corresponds to an event. Therefore, a higher HR (>1) corresponds to faster RTW.

Results were expressed as hazard ratios (HRs) with 95% confidence intervals (CIs). STATA (StataCorp. 2025. Stata Statistical Software: Release 19. StataCorp LLC, College Station, TX, USA) was used for the statistical analysis.

## 3. Results

### 3.1. Patient Characteristics

A total of 103 patients were enrolled, with a mean age of 56.8 ± 7.2 years; 92.2% were male. Current smokers accounted for 42.7% of the cohort, and 29.1% were former smokers. BMI was 26.7 ± 3.6 kg/m^2^; 41.8% were overweight and 20.4% obese. Hypertension (65%), dyslipidemia (74.8%), and diabetes mellitus (14.6%) were the most frequent risk factors. A positive family history of CV disease was present in 26.2% of participants (Table 1).

Regarding clinical presentation and procedures, as shown in Table 2, 69.9% of patients were admitted for acute coronary syndrome (ACS), including STEMI (43.7%) and NSTEMI (26.2%). PCI was performed in 64.1% of cases, CABG in 18.5%, and valve replacement or thoracic aortic surgery in 15.5%. Out-of-hospital cardiac arrest (OHCA) before admission occurred in 9 patients.

Mean LVEF at admission was 55.8 ± 9.2%, improving to 57.4 ± 6.8% post-treatment. Heart failure was diagnosed in 14.6% of patients. Periprocedural complications included arrhythmias (38.8%), anemia (30.1%), and renal impairment (49.5%). Arrhythmias persisted in 32.0% of cases (5 atrial fibrillation and 27 frequent ventricular premature beats).

Overall, 87.4% completed CR. After discharge, 36.4% of smokers quit and 74.1% of CAD patients reached LDL targets. Quality of life was high, with a mean EQ-5D index of 0.92 ± 0.09 and EQ-VAS score of 77.4 ± 12.9. Twelve patients accessed ED after discharge but only 2 had a cardiovascular related condition (one had uncontrolled hypertension, the other a symptomatic arrhythmia). Among other 10 patients, only one self-reported an anxiety score equal to 5 at EQ-5D questionnaire. All these 10 patients, who accessed ED for suspected cardiovascular related problems, were discharged from ED without a diagnosed clinical condition and without any prescription.

When comparing patients who completed CR with those who did not, no major clinical differences were observed apart from LVEF. At discharge, median LVEF was similar between groups (56.5% in CR vs. 57.0% in non-CR), and between-group differences at follow-up also remained negligible. However, when testing LFEV differences before and after (at admission vs. post-discharge), a clear improvement was observed only among participants who completed CR (56.6% at admission vs. 58% post-discharge; *p* = 0.008), whereas no significant differences were found in non-CR participants (*p* = 0.61).

### 3.2. Occupational Characteristics

Before admission, 95.2% of participants were considered fit for work without restrictions, with a mean perceived work ability of 8.8 ± 1.8 (median 9.0). Half of the cohort were white-collar workers (50.5%) and the remainder blue-collar (49.5%).

**Figure 1:**
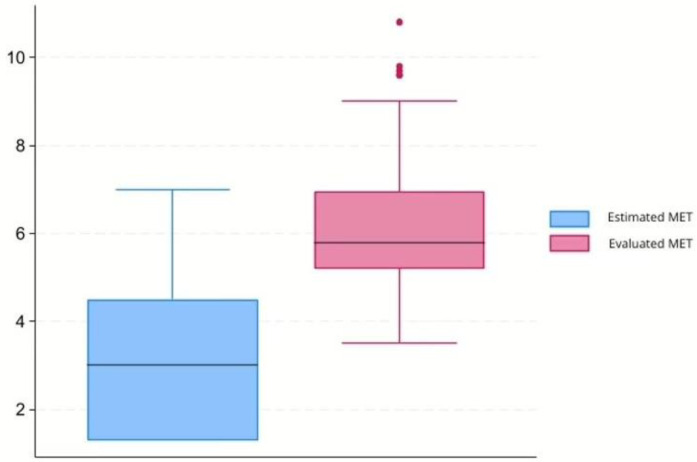
Comparison between METs, metabolic equivalents of task estimated for the specific task according to Compendium of Physical Activities [16], and METs assessed with the stress test.

Occupational workload was classified as light (<3 METs) in 48.5%, moderate (3–6 METs) in 35.0%, and heavy (>6 METs) in 16.5%. The mean workload estimated by functional testing (6.19 ± 1.54 METs) was higher than that estimated from job tasks (3.26 ± 2.01 METs), suggesting a systematic overestimation of occupational demands, as shown in Figure 1.

Most participants (72.8%) had roles of responsibility. Self-employment accounted for 61.2% of cases, and 21.4% performed shift work. Full-time employment was reported by 85.4%.

A detailed overview of occupational characteristics is summarized in Table 2.

### 3.3. Return to Work and Functional Outcomes

During the follow-up period, 80 patients (77.7%) returned to work, whereas 23 (20.8%) did not resume employment (censored). Table 3 displays the distribution of workers by return to work status and further sub-categories:

Among 80 patients returning to work, only one changed his job-task. Among 79 who retrieved the same job-task, 22 received new occupational restrictions, as follows:

- A total of 19 restrictions on manual handling of loads;
- A total of 4 changed from full to part time contract;
- A total of 5 were restricted from night shifts.

Among 23 patients who did not return to work:

- A total of 5 were close to retirement, hence they extended their convalescence until pension.
- A total of 4 were included in re-employment lists reserved for disable people (by Italian law 68/1999).
- The contract of 3 workers, ending during their convalescence, was not renewed after expiration.
- Evaluation of fitness of work was still pending for 11 workers.

Among those who returned to work, the mean time to RTW was 58.9 ± 47.0 days. Main reasons for non-RTW were retirement, mismatch between physical capacity and job requirements, or pending occupational reassignment.

Self-perceived work ability decreased slightly post-event (mean 8.3 ± 1.4; median 8.0). According to WAI categories, 46.6% reported good, 19.4% moderate, 6.8% poor, and 4.9% excellent work ability.

### 3.4. Predictors of Return to Work

Associations between patient characteristics and reemployment status are summarized in Supplementary Table S1. After the backward selection procedure, following predictors were selected in our final models: age, occupational physical demand, responsibility at work, job contract (self-employed vs. employed), CR, PCI and Arrhythmia post discharge (Table 4).

In unadjusted analyses, several clinical and occupational factors were strongly associated with return to work (RTW). Specifically, PCI (49.6 ± 52.7; *p* = 0.021), self-employment (34.8 ± 28.2; *p* < 0.001), having a role of responsibility (47.4 ± 42.0; *p* < 0.001), and lower physical job demands (42.0 ± 33.2; *p* = 0.004) were associated with reemployment, whereas shift work (91.1 ± 57.8; *p* = 0.019), participation in CR (64.3 ± 47.6; *p* = 0.005) and having undergone more complex procedures such as combined valve and thoracic aorta surgery (121.6 ± 59.1; *p* = 0.012), compared to valve surgery alone, were linked to delayed or absent RTW.

Sex and major comorbidities were not related to reemployment. Among continuous variables, both WAI (Spearman ρ = −0.27, *p* = 0.015) and EQ-VAS (ρ = −0.25, *p* = 0.025) were positively associated with earlier RTW. LVEF showed no association with RTW (ρ = −0.07, *p* = 0.523).

Time-to-event analyses using Cox proportional hazards models are presented in Table 4.

In the final multivariate Cox regression model, self-employment was strongly associated with an earlier return to work (HR = 5.08, 95% CI 2.52–10.27), as was having occupational responsibility, which was associated with approximately twice the probability of returning to work compared with those without responsibility at work (HR = 2.12, 95% CI 1.01–4.45). On the other hand, higher physical demand at work was associated with a delayed return to work, although this association was borderline.

About clinical predictors, patients who underwent PCI had a 2.7-fold higher probability of an earlier RTW compared with those treated with surgery (HR = 2.72, 95% CI 1.47–5.06). Conversely, both underwent cardiac rehabilitation and having arrhythmia post discharge showed a suggestive association of a higher probability of delayed RTW (Table 4).

Similarly, time-to-event analyses using Cox proportional hazards models were conducted in male patients only, and the results were largely comparable to those of the overall cohort (Table 5).

As sensitivity analysis, a Cox regression model restricted to patients with CAD was fitted, showing comparable findings (Supplementary Table S2).

## 4. Discussion

This prospective cohort study explored the multifactorial determinants of RTW after CV events in a working-age population. With an overall RTW rate of 77.7% and an average resumption time of two months, our findings are consistent with international data [5]. Importantly, they also highlight key clinical, psychosocial, and occupational variables that shape reintegration trajectories.

### 4.1. Sociodemographic Factors

The sociodemographic profile of our cohort provides meaningful insight into RTW after CV events. The population was predominantly male, reflecting both the higher incidence of ischemic heart disease among men [21]. The marked male predominance is also consistent with national labour statistics: in Italy, female participation in the labour market declines substantially in the years approaching retirement, with an employment rate of approximately 35% among women aged 60–64, markedly lower than that of men in the same age group [22]. Moreover, CV disease typically manifests at an older age in women than in men [23], and many women have already exited the workforce by the time such events occur. This combination of later disease onset and earlier workforce withdrawal makes it inherently challenging to recruit a sex-balanced sample of working-age patients with recent cardiovascular disease. Consequently, female representation was limited, precluding a detailed analysis of sex-specific differences in psychological adjustment and quality of life, which are frequently reported as determinants of RTW in the literature [14]. Sensitivity analyses excluding women yielded the same results, supporting the robustness of our findings.

Age emerged as a complex, multifactorial variable. In the final multivariate model, age was not related with time to RTW. This may reflect that older individuals were more frequently self-employed, affording them greater flexibility and stronger financial motivation, and were often engaged in occupations with lower physical demands, as indicated by reduced estimated metabolic workloads (METs). Conversely, participants nearing retirement often did not return to work at all, suggesting that age influences both the timing and likelihood of reintegration. These observations contrast with most prior studies, where older age consistently predicted delayed or reduced RTW after myocardial infarction, as summarized in the review by Sun et al. [24]. Together, these findings underscore the need to interpret the role of age in the context of occupational and socioeconomic factors.

### 4.2. Clinical Factors

From a clinical standpoint, most patients presented with preserved or mildly reduced left ventricular function and low rates of adverse outcomes such as heart failure or recurrent ischemia. Participation in cardiac rehabilitation (CR) pathway was high (87.4%), consistent with guideline recommendations [25]. Interestingly, patients who completed CR returned to work later—a finding likely explained by selection bias, since CR is typically prescribed for patients with greater clinical complexity or lower baseline autonomy. Similar results were reported in a Danish registry, where Pedersen et al. observed lower RTW rates among CR participants at three months but higher rates at nine to twelve months, reflecting delayed yet improved long-term reintegration [26]. Early delays may thus reflect baseline differences and the progressive nature of CR programs, which prioritize safe recovery over rapid return.

In our cohort, clinical parameters were largely comparable between CR participants and non-participants except for LVEF. Although discharge values were similar, only CR participants showed improvement at follow-up, supporting the role of CR in functional recovery, even if this did not translate into earlier RTW. Longer follow-up is needed to capture CR’s full impact on work capacity and psychosocial well-being.

CR is multidisciplinary, including exercise training, education, dietary counseling, smoking cessation, and psychological support [19]. In our cohort, 36.4% of smokers quit and 74.1% achieved LDL targets, confirming effective pharmacological control but highlighting the challenge of sustained lifestyle change. Given the elevated BMI, future studies should assess whether CR supports long-term weight management.

Heart failure was not related to RTW duration, possibly due to the small number of cases or selection bias, as patients with advanced disease were less likely to be employed or return within the study window. Similarly, neither recurrent events nor residual stenosis were associated with prolonged absence. LVEF tended to be higher in those who returned earlier, suggesting that preserved cardiac function may facilitate reintegration but interacts with psychosocial and occupational factors [5,20].

Comorbidities did not delay RTW overall. Nonetheless, as highlighted by the European Society of Cardiology [5], conditions such as diabetes, renal failure, prior stroke, and peripheral artery disease affect both physical capacity and reintegration potential. These comorbidities often correspond to pre-existing or newly introduced work restrictions, many already in place before the acute event.

The type of CV intervention also influenced RTW. Patients undergoing PCI—a less invasive procedure—returned earlier than those treated with CABG or valve surgery, consistent with published data [20]. Combined valve and aortic surgery was associated with a longer period of absence from work, reflecting the additive burden of complex interventions.

Complications such as anemia and renal impairment were common but transient and had minimal impact on RTW. In contrast, arrhythmias delayed reintegration, likely due to persistent symptoms, the need for monitoring, or safety restrictions in high-risk jobs (e.g., driving or work at heights). Although the prognostic significance of atrial fibrillation differs from that of frequent ventricular ectopy, in occupational medicine the persistence of any rhythm disorder—regardless of subtype—tends to result in similar work limitations. Occupational physicians often apply precautionary restrictions when arrhythmias are still present, particularly in safety-sensitive roles, which may explain their uniform impact on RTW in our cohort. This underscores the importance of close collaboration between cardiologists and occupational physicians: clearer, task-specific recommendations from cardiology specialists could support more accurate fitness-for-duty assessments and help avoid unnecessarily restrictive decisions when no contraindications to specific work activities are present. Although excluded from Cox models, out-of-hospital cardiac arrest (OHCA) remains clinically relevant given its variable outcomes. While OHCA is often associated with poorer functional recovery, in our cohort only one patient developed neurological sequelae limiting RTW. Because follow-up was calculated from the date of hospital discharge, differences in acute-phase recovery did not influence RTW analyses. For this reason, OHCA survivors were included in the study, and the Cox model was stratified by OHCA status to account for differences in baseline hazards.

Despite good objective recovery, subjective assessments revealed residual vulnerability. The Work Ability Index (WAI) declined slightly (median 9.0 to 8.0), indicating that fear of recurrence and reduced confidence may limit perceived capacity. Patients who returned earlier reported higher WAI and EQ-VAS scores, underscoring the influence of self-perception on reintegration.

Although overall quality of life of patients was high, some without overt anxiety repeatedly accessed ED after discharge without any confirmed cardiovascular condition. Since EQ-5D score is self-reported, it may not be optimal to identify subjective conditions like anxiety/depression. Therefore, process measures such as inappropriate ED access may be a useful integrative indicator to screen mental health conditions requiring psychological support. There is already a body evidence of association between inappropriate ED accesses for cardio-vascular conditions and anxiety in the open literature [27,28].

Targeted psychological support could improve well-being, reduce unnecessary healthcare use, and facilitate sustainable RTW.

### 4.3. Occupational Factors

Occupational characteristics strongly influenced RTW patterns. Lower physical workload was associated with earlier reintegration, whereas moderate-to-high job demands delayed RTW. This finding is consistent with prior studies showing that occupational physical strain—unlike leisure-time exercise—may increase CV burden, known as the “physical activity paradox” [29].

Self-employment was the strongest predictor of early RTW. Financial pressure and greater job flexibility likely explain this finding, though it also raises concerns about the lack of structured occupational health surveillance for self-employed workers. Tailored follow-up and wearable monitoring technologies could help reduce risks in this population.

Functional evaluations, including exercise stress testing, proved particularly valuable for assessing CV adaptation to job demands and for reassuring both patients and occupational physicians regarding fitness for work. Work-related risks such as shift work, work at heights, or exposure to electromagnetic fields require individualized evaluation.

Central to the reintegration process was structured counselling delivered jointly by cardiologists and occupational physicians. After completing functional assessments, patients received individualized exercise prescriptions, including recommended training intensities and, when appropriate, the use of wearable devices to monitor clinical parameters. This approach was particularly relevant for self-employed workers who are not covered by formal occupational health surveillance. Occupational physicians provided guidance on safe modalities for work resumption, and when incompatibilities between clinical status and job tasks were identified, patients were assisted in initiating change in job task or referral to targeted reassignment programs (Italian Law 68/1999).

Patients with managerial or decision-making roles returned earlier than their counterparts. In addition to lighter physical demands, this may reflect stronger professional identity, higher motivation, or employer-driven flexibility. Only one patient changed job role, highlighting the underuse of task modifications or reassignments despite their potential to facilitate reintegration.

After reintegration, 22 new task-specific restrictions were introduced. Monitoring their evolution is important within existing regulatory frameworks. For instance, night shifts are typically restricted for one year after an acute event, and high-risk occupations are usually deferred until completion of dual antiplatelet therapy (commonly 12 months). These considerations emphasize the importance of ongoing occupational health surveillance.

Although shift work was not associated with a change in time to RTW in our final models, it remains a well-established CV risk factor and practical barrier to reintegration, particularly in patients with arrhythmias or residual ischemia [30]. Similarly, long working hours were not predictive in this cohort, possibly due to confounding by other occupational variables.

In addition, 22 patients (22.3%) did not return to work during follow-up. The main reasons included approaching retirement, persistent symptoms, low perceived work ability, or a mismatch between functional capacity and job demands—often related to safety-sensitive tasks. In several cases, ongoing work-fitness evaluations or regulatory constraints contributed to delayed reintegration. These findings highlight the substantial occupational impact of CV disease: even when clinical recovery is satisfactory, residual limitations and workplace safety requirements may hinder full reintegration. The proportion of non-RTW patients in our cohort aligns with published data [5], confirming comparability with existing evidence.

Overall, these findings underscore the pivotal role of occupational medicine in balancing job-specific demands with individual health, performing risk assessments, and developing tailored RTW strategies. In the context of aging workforces and increasing multimorbidity, regular reassessment of work capacity is essential to sustain employability and protect CV health.

### 4.4. Strengths and Limitations

The strengths of this study include its prospective design, integration of the main clinical, psychosocial, and occupational variables, and use of validated instruments such as the WAI and EQ-5D. Importantly, functional testing was tailored to occupational demands, providing ecologically valid assessments of work capacity. In addition, several occupational and clinical covariates were tested and included in the final analysis to evaluate their contribution to the prediction of time to return to work.

Limitations must also be acknowledged. The single-centre design and relatively small sample size may limit results. Although a wide range of clinical and functional variables was initially considered, the total number of patients was limited. Although the number of events relative to the number of predictors in the final model was acceptable (80 events for 7 predictors, corresponding to approximately 11 events per variable), some risk of overfitting cannot be completely excluded. The model was developed in a single cohort with a modest sample size, and hazard ratios may therefore be sensitive to random variation. Consequently, some associations may remain undetected or imprecisely estimated. The modest sample size restricts the ability to explore more complex relationships between predictors. However, to mitigate the risk of model overfitting under these conditions, we applied a backward selection approach, retaining only covariates that showed meaningful associations with the outcome in the final Cox models. Nonetheless, caution is warranted when interpreting the results. Given these considerations, the present study should be regarded as a pilot investigation, intended to generate hypotheses and guide future, more powered research. Another limitation is given by the strong predominance of male patients prevented detailed analysis of sex-specific factors. Furthermore, the follow-up duration was relatively short, precluding evaluation of long-term sustainability of employment or recurrent CV events. Finally, although CR was associated with delayed RTW, longer follow-up is required to capture its full benefits.

A further limitation concerns the heterogeneity of the study population, which included patients with different types of CV events and procedures. Although this reflects real-world clinical practice in occupational medicine, such variability may reduce comparability across subgroups and affect the precision of estimates. To mitigate the impact of this heterogeneity, and because some clinical characteristics did not satisfy the proportional hazards assumption, we adopted a stratified Cox model to account for differences in baseline hazard functions. In addition, sensitivity analyses restricted to ACS-only patients yielded results consistent with the main findings, supporting the robustness of the observed associations (Table S2). Nonetheless, the heterogeneous nature of the sample must be considered when interpreting the results, and future larger studies should investigate more homogeneous clinical subgroups. Despite these methodological adjustments, the heterogeneous nature of the cohort remains an intrinsic limitation, and future studies with larger and more homogeneous samples are warranted to explore event-specific predictors of return to work.

## 5. Conclusions

This study highlights the complex and multifactorial nature of RTW following CV events. In our working-age cohort, nearly four out of five patients resumed employment within two months, a rate comparable with international evidence. Beyond clinical recovery, occupational and psychosocial factors emerged as critical determinants of RTW.

Less invasive procedures (PCI), managerial responsibility, and self-employment facilitated earlier reintegration, whereas high physical job demands, persistent arrhythmias, and participation in structured cardiac rehabilitation were associated with slower return. The latter likely reflects selection of more clinically fragile patients into rehabilitation pathways, with long-term benefits that may outweigh early delays.

These findings emphasize that RTW cannot be considered solely a marker of clinical recovery. Effective reintegration requires coordinated input from cardiologists, occupational physicians, and employers. Tailored interventions—including workplace accommodations, functional assessments, and psychosocial support—are essential to sustain RTW and quality of life, particularly in the context of an aging workforce.

Future research should expand follow-up duration and explore the role of digital health tools in supporting safe and sustainable RTW after CV events.

## Figures and Tables

**Table 1:** Sociodemographic data and CV risk factors.

Characteristics	
Patients, n (%)	103.0 (100.0)
Sex, n (%)	
Females	8.0 (7.8)
Males	95.0 (92.2)
Age (years), mean ± SD (median)	56.8 ± 7.2 (59.0)
Smoking, n (%)	
Never-smokers	29.0 (28.2)
Smokers	44.0 (42.7)
Ex-smokers	30.0 (29.1)
BMI (kg/m^2^), mean ± SD (median)	26.7 ± 3.6 (26.3)
Normal, n (%)	39.0 (37.9)
Overweight, n (%)	43.0 (41.8)
Obese, n (%)	21.0 (20.4)
Hypertension, n (%)	67.0 (65.0)
Diabetes, n (%)	15.0 (14.6)
Dyslipidemia, n (%)	77.0 (74.8)
Previous CV event, n (%)	25.0 (24.3)
Family history of premature IHD, n (%)	27.0 (26.2)

BMI, body mass index; CV, cardiovascular; IHD, ischemic heart disease.

**Table 2:** Occupational and clinical admission characteristics of the study population.

Characteristics	
**Clinical profile**	
Reason of the admission, n (%)	
CAD	87.0 (84.5)
ACS	72.0 (69.9)
STEMI	45.0 (43.7)
NSTEMI	27.0 (26.2)
VHD	16.0 (15.5)
Type of intervention, n (%)	
PCI	66.0 (64.1)
CABG	19.0 (18.5)
VR	15.0 (14.6)
Aortic surgery	7.0 (6.8)
LVEF at admission, mean ± SD (median)	55.8 ± 9.2 (57.0)
Heart failure, n (%)	15.0 (14.6)
Arrhythmia at admission, n (%)	
NSVT	23.0 (22.3)
AF	17.0 (16.5)
Arrhythmia post discharge, n (%)	33.0 (32.0)
Anemia, n (%)	31.0 (30.1)
Renal failure * (mild or more), n (%)	51.0 (49.5)
Residual critical ** stenosis, n (%)	12.0 (11.7)
Therapy, n (%)	
Beta-Blocker	86 (83.5)
ACE-inhibitor/sartan	76 (73.8)
Acetylsalicylic acid	90 (87.4)
DAPT	83 (80.6)
NOAC	7.0 (6.80)
Statin	97.0 (94.2)
Ezetimibe	93.0 (90.3)
Bempedoic acid	8.0 (7.8)
PCSK9 Inhibitor	4.0 (3.9)
Metformin	15.0 (14.6)
SGLT2 inhibitor	15.0 (14.6)
GLP-1 agonists	2.0 (1.9)
Cardiac rehabilitation, n (%)	90.0 (87.4)
LVEF post-discharge, mean ± SD (median)	57.4 ± 6.8 (58.0)
Occupational profile	
Occupation, n (%)	
Blue Collar workers	51.0 (49.5)
White Collar workers	52.0 (50.5)
Occupational physical demand ***, n (%)	
Light	50.0 (48.5)
Moderate	36.0 (35)
Vigorous	17.0 (16.5)
Responsibility at work, n (%)	75.0 (72.8)
Job contract, n (%)	
Self-employed worker	63.0 (61.2)
Employee	40.0 (38.8)
Working hours, n (%)	
Full-time	88.0 (85.4)
Part-time	15.0 (14.6)
Shift work, n (%)	21.0 (21.4)
Occupational exposure risks, n (%)	
VDT	41.0 (39.8)
MHL	47.0 (45.6)
Microclimate	23.0 (22.3)
Noise	34.0 (33.0)
Vibration	23.0 (22.3)
Work at height	21.0 (20.4)
Confined spaces	9.0 (8.7)
EMF	6.0 (5.8)
Night shift	12.0 (11.7)
Other	8.0 (7.8)
Fit for work—before, n (%)	
Without restrictions	98.0 (95.2)
With restrictions	5.0 (4.9)
Perceived work ability 0–10 before, mean ± SD (median)	8.8 ± 1.8 (9.0)
RTW, n (%)	80.0 (77.7)
Duration of Absence (days), mean ± SD (median)	58.9 ± 47.0 (45.0)
Same job after RTW, n (%)	79.0 (98.8)
New work restrictions, n (%)	22.0 (27.5)
Perceived work ability 0–10 after, mean ± SD (median)	8.3 ± 1.4 (8.0)
Work Ability Index (WAI), mean ± SD	37.2 ± 5.1 (38.0)
Poor (7–27), n (%)	7.0 (6.8)
Moderate (28–36), n (%)	20.0 (19.4)
Good (37–43), n (%)	48.0 (46.6)
Excellent (44–49), n (%)	5.0 (4.9)
EQ-5D-5L, mean ± SD	
EQ-5D descriptive system	0.92 ± 0.09 (1.0)
EQ visual analogue scale (EQ VAS)	77.4 ± 12.9 (80.0)

CAD, coronary artery disease; ACS, Acute Coronary Syndrome; STEMI, ST-segment elevation myocardial infarction; NSTEMI, non-ST-segment elevation myocardial infarction; VHD, valvular heart disease; PCI, percutaneous coronary intervention; CABG, coronary artery bypass graft; VR, valve replacement; LVEF, left ventricular ejection fraction; NSVT, No sustained ventricular tachycardia; AF, atrial fibrillation; ACE, Angiotensin-converting enzyme; DAPT, Dual Antiplatelet Therapy; NOAC, Non-vitamin K antagonist oral anticoagulant; PCSK9, Protein Convertase Subtilisin/Kexin type 9; SGLT2, Sodium–Glucose Transport Protein 2 (SGLT2) Inhibitors; GLP-1, Glucagon-like peptide-1. * eGRF < 90 mL/min/1.73 m^2^ according to CKD-EPI equation. ** Residual critical stenosis is defined as a ≥50% stenosis in the coronary artery. VDT, visual display terminal; MHL, manual handling of loads; EMF, electromagnetic field; RTW, Return to work. *** Occupational physical demands were classified based on estimated energy expenditure for specific tasks, according to the Compendium of Physical Activities [16].

**Table 3:** Frequency distribution of workers by return to work after discharge and further sub-categories.

Return to Work	Sub-Categories			
Yes (n = 80)	Change in job-task (n = 1)			
	Returned to same job (n = 79)	Novel occupational restrictions	No (n = 57)	
			Yes (n = 22)	Manual handling of loads (n = 19)
				Part time contract (n = 4)
				Night shifts (n = 5)
No (N = 23)	Contract not renewed (n = 3)			
	Inclusion in disability list (n = 4)			
	Retirement (n = 5)			
	Pending FTW evaluation (n = 11)			

FTW = Fitness to work.

**Table 4:** Cox proportional hazards model for earlier RTW. Analyses were based on 8444 person-days of observation.

	Univariate		Bivariate (Adjusted for Age)		Multivariate *	
Variable	HR (95% CI)	*p* Value	HR (95% CI)	*p* Value	HR (95% CI)	*p* Value
Age	1.03 (1.00–1.07)	0.054			0.99 (0.95–1.03)	0.466
Occupational physical demand						
Moderate	0.55 (0.34–0.90)	0.017	0.58 (0.35–0.95)	0.029	0.54 (0.28–1.02)	0.057
Vigorous	0.38 (0.19–0.76)	0.006	0.41 (0.20–0.82)	0.011	0.43 (0.17–1.06)	0.066
Responsibility at work	2.90 (1.67–5.05)	0.000	2.99 (1.71–5.23)	0.000	2.12 (1.01–4.45)	0.048
Self-Employed	4.12 (2.53–6.71)	0.000	3.95 (2.42–6.47)	0.000	5.08 (2.52–10.27)	0.000
Cardiac Rehabilitation	0.40 (0.21–0.75)	0.004	0.41 (0.22–0.76)	0.005	0.46 (0.20–1.06)	0.068
PCI	1.40 (0.88–2.24)	0.155	1.44 (0.90–2.30)	0.130	2.72 (1.47–5.06)	0.002
Arrhythmia post discharge	0.73 (0.42–1.27)	0.268	0.69 (0.40–1.21)	0.195	0.56 (0.28–1.09)	0.088

PCI, percutaneous coronary intervention; * results of final multivariate model, stratified by sex, smoking status, out-of-hospital cardiac arrest and heart failure, to account for differences in baseline hazards.

**Table 5:** Cox proportional hazards model for earlier RTW in male patients. Analyses were based on 7842 person-days of observation.

	Univariate		Bivariate (Adjusted for Age)		Multivariate *	
Variable	HR (95% CI)	*p* Value	HR (95% CI)	*p* Value	HR (95% CI)	*p* Value
Age	1.03 (1.00–1.06)	0.097			0.98 (0.94–1.02)	0.407
Occupational physical demand						
Moderate	0.59 (0.35–0.99)	0.046	0.61 (0.36–1.03)	0.063	0.56 (0.29–1.07)	0.081
Vigorous	0.40 (0.20–0.80)	0.010	0.42 (0.21–0.85)	0.016	0.43 (0.17–1.07)	0.069
Responsibility at work	3.18 (1.76–5.73)	0.000	3.27 (1.81–5.93)	0.000	2.35 (1.09–5.06)	0.030
Self-Employed	4.11 (2.47–6.83)	0.000	3.97 (2.38–6.63)	0.000	5.12 (2.52–10.37)	0.000
Cardiac Rehabilitation	0.43 (0.21–0.87)	0.018	0.44 (0.22–0.89)	0.023	0.50 (0.21–1.20)	0.122
PCI	1.42 (0.87–2.31)	0.160	1.44 (0.89–2.35)	0.140	2.86 (1.51–5.42)	0.001
Arrhythmia post discharge	0.67 (0.37–1.20)	0.177	0.65 (0.36–1.16)	0.146	0.54 (0.28–1.06)	0.075

PCI, percutaneous coronary intervention; * results of final multivariate model, stratified by smoking status, out-of-hospital cardiac arrest and heart failure, to account for differences in baseline hazards.

## Data Availability

Data are not publicly available, since they were purposively collected by the authors for the present study, but are available from the corresponding author or reasonable request.

## Supplementary Materials

The following supporting information can be downloaded at: https://www.mdpi.com/article/10.3390/life16010019/s1, Table S1: Characteristics and association with reemployment; Table S2: Cox Proportional Hazards Model for Earlier RTW. Analyses were based on 6833 person-days of observation and restricted to patients with Coronary Artery Disease (CAD) only (n = 87).

## Abbreviations

The following abbreviations are used in this manuscript:

RTW	Return to work
CVD	Cardiovascular disease
STEMI	ST-segment elevation myocardial infarction
NSTEMI	Non-ST-segment elevation myocardial infarction
CR	Cardiac Rehabilitation
PCI	Percutaneous coronary intervention
CABG	Coronary artery bypass grafting
BMI	Body mass index
CCI	Charlson comorbidity index
LVEF	Left ventricular ejection fraction
METs	Metabolic equivalants
WAI	Work ability index
HRQoL	Health-related quality of life
VAS	Visual analogue scale
