# Supplementary material for: Optimising Return to Work for Cardiovascular Patients: An Interdisciplinary Approach in Occupational Medicine and Cardiology
Source: Life (Basel). 2025 Dec 22;16(1):19. doi: 10.3390/life16010019 (PMC12842632; doi:10.3390/life16010019)
Supplement: Supplementary file 1 [file life-16-00019-s001.zip › life-4036150-supplementary.pdf]

**Supplementary Table S1.** Characteristics and association with reemployment.

| Variables | | Duration of absence | | |
|---|---|---|---|---|
| | | **Mean ± SD** | **Median** | **p value*** |
| **Sex** | Females | 63.7 ± 52.8 | 60.0 | 0.681 |
| | Males | 58.5 ± 46.8 | 45.0 | |
| **OHCA** | Yes | 62.4 ± 57.5 | 60.0 | 0.979 |
| | No | 58.6 ± 46.3 | 45.0 | |
| **Smoking** | Never-smokers | 58.6 ± 48.6 | 45.0 | 0.822** |
| | Smokers | 60.7 ± 43.1 | 60.0 | |
| | Ex-smokers | 57.4 ± 51.0 | 52.5 | |
| **Diabetes** | Yes | 58.3 ± 48.8 | 45.0 | 0.396 |
| | No | 62.7 ± 35.2 | 60.0 | |
| **BMI** | Normal | 64.4 ± 48.6 | 52.5 | 0.630 |
| | Overweight | 53.3 ± 43.6 | 40.0 | |
| | Obese | 62.0 ± 53.0 | 52.5 | |
| **STEMI** | Yes | 52.1 ± 41.7 | 37.5 | 0.087 |
| | No | 74.8 ± 55.3 | 60.0 | |
| **NSTEMI** | Yes | 58.3 ± 45.7 | 40.0 | 0.907 |
| | No | 59.1 ± 47.9 | 60.0 | |
| **PCI** | Yes | 49.6 ± 52.7 | 60.0 | **0.021** |
| | No | 76.2 ± 52.7 | 32.5 | |
| **CABG** | Yes | 73.2 ± 41.3 | 60.0 | 0.075 |
| | No | 55.9 ± 47.8 | 40.0 | |
| **VHD** | VS | 77.6 ± 48.1 | 75.0 | **0.012** |
| | Aortic surgery | 121.6 ± 59.1 | 98.0 | |
| **Anemia** | Yes | 70.0 ± 55.1 | 60.0 | 0.491 |
| | No | 56.1 ±43.5 | 45.0 | |
| **Heart failure** | Yes | 69.6 ± 60.1 | 67.5 | 0.512 |
| | No | 57.0 ±44.6 | 45.0 | |
| **Previous CV events** | Yes | 44 ± 36.1 | 30.0 | 0.102 |
| | No | 64.2 ± 49.5 | 60.0 | |
| **Arrhythmia post-discharge** | Yes | 70.1 ± 50.2 | 60.0 | 0.136 |
| | No | 53.5 ± 44.8 | 42.5 | |
| **Cardiac rehabilitation** | Yes | 64.3 ± 47.6 | 60.0 | **0.005** |
| | No | 28.6 ± 29.4 | 14.0 | |
| **Occupational physical demand** | Light | 42.0 ± 33.2 | 30.0 | **0.004**** |
| | Moderate | 76.4 ± 53.3 | 70.0 | |
| | Vigorous | 84.5 ± 54.6 | 90.0 | |
| **Responsibility at work** | Yes | 47.4 ± 42.0 | 32.5 | **0.000** |
| | No | 105 ± 37.1 | 95.0 | |
| **Job contract** | Self-employee | 34.8 ± 28.2 | 27.0 | **0.000** |
| | Employee | 80.7 ± 50.1 | 90.0 | |
| **Working hours** | Full-time | 58.9 ± 47.6 | 45.0 | 0.822 |
| | Part-time | 58.9 ± 45.0 | 60.0 | |
| **Shift work** | Yes | 91.1 ± 57.8 | 45.0 | **0.019** |
| | No | 52.1 ± 41.8 | 95.0 | |
| **Work Ability Index (WAI)** | Poor | 67.1 ± 73.7 | 30.0 | 0.088** |
| | Moderate | 71.1 ± 47.9 | 65.0 | |
| | Good | 57.0 ± 42.7 | 52.5 | |
| | Excellent | 17.4 ± 10.4 | 15.0 | |

OHCA, out of hospital cardiac arrest; BMI, body mass index; STEMI, ST-segment elevation myocardial infarction; NSTEMI, non-ST-segment elevation myocardial infarction; PCI, percutaneous coronary intervention; CABG, coronary artery bypass graft; VHD, valvular heart disease; CV, cardiovascular. *Mann-Whitney test; ** Kruskal–Wallis test

**Table S2.** Cox Proportional Hazards Model for Earlier RTW. Analyses were based on 6,833 person-days of observation and restricted to patients with Coronary Artery Disease (CAD) only (N = 87)

| | **Univariate** | | **Bivariate (adjusted for age)** | | **Multivariate** | |
|---|---|---|---|---|---|---|
| **Variable** | **HR (95% CI)** | **p value** | **HR (95% CI)** | **p value** | **HR (95% CI)** | **p value** |
| **Age** | 1.03 (0.99 - 1.07) | 0.111 | -- | -- | 0.98 (0.94 - 1.03) | 0.431 |
| **Occupational physical demand** | | | | | | |
| **Moderate** | 0.53 (0.31 – 0.91) | 0.022 | 0.54 (031 – 0.93) | 0.027 | 0.43 (0.21 – 0.89) | 0.023 |
| **Vigorous** | 0.39 (0.18 – 0.84) | 0.016 | 0.41 (0.19 – 0.89) | 0.024 | 0.48 (0.18 - 1.26) | 0.138 |
| **Responsibility at work** | 3.52 (1.87 – 6.66) | 0.000 | 3.56 (1.88 – 6.74) | 0.000 | 2.67 (1.05 - 6.75) | 0.038 |
| **Self-Employed** | 4.84 (2.81 – 8.33) | 0.000 | 4.69 (2.72 – 8.11) | 0.000 | 5.61 (2.59 -12.15) | 0.000 |
| **Cardiac Rehabilitation** | 0.42 (0.22 – 0.79) | 0.007 | 0.42 (0.22 – 0.79) | 0.007 | 0.51 (0.21 - 1.24) | 0.137 |
| **PCI** | 1.37 (0.77 – 2.45) | 0.286 | 1.48 (0.82 – 2.67) | 0.190 | 2.61 (1.21 - 5.62) | 0.014 |
| **Arrhythmia post discharge** | 0.67 (0.36 – 1.25) | 0.208 | 0.61 (0.32 – 1.15) | 0.127 | 0.44 (0.20 – 0.96) | 0.040 |

PCI, percutaneous coronary intervention.
